# 3-(3-Chloro­phenyl­sulfon­yl)-5-isopropyl-2-methyl-1-benzofuran

**DOI:** 10.1107/S1600536812008525

**Published:** 2012-03-03

**Authors:** Hong Dae Choi, Pil Ja Seo, Uk Lee

**Affiliations:** a Department of Chemistry, Dongeui University, San 24 Kaya-dong Busanjin-gu, Busan 614-714, Republic of Korea; b Department of Chemistry, Pukyong National University, 599-1 Daeyeon 3-dong, Nam-gu, Busan 608-737, Republic of Korea

## Abstract

In the title compound, C_18_H_17_ClO_3_S, the 3-chloro­benzene ring makes a dihedral angle of 82.04 (5)° with the mean plane [r.m.s. deviation = 0.006 (1) Å] of the benzofuran fragment. In the crystal, mol­ecules are linked by weak C—H⋯O and C—H⋯π inter­actions.

## Related literature

For background information and the crystal structures of related compounds, see: Choi *et al.* (2010 ▶, 2011 ▶).
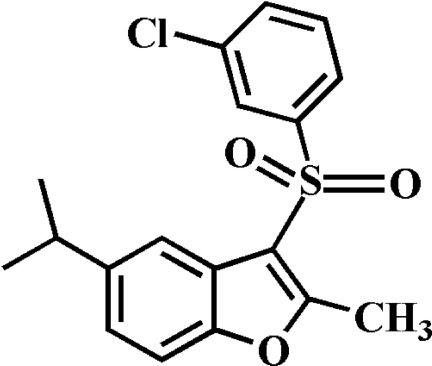


## Experimental

### 

#### Crystal data

C_18_H_17_ClO_3_S
*M* *_r_* = 348.83
Triclinic, 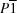

*a* = 7.1700 (2) Å
*b* = 9.9400 (2) Å
*c* = 12.2508 (3) Å
α = 83.484 (1)°
β = 77.907 (1)°
γ = 85.707 (1)°
*V* = 847.05 (4) Å^3^
*Z* = 2
Mo *K*α radiation
μ = 0.36 mm^−1^
*T* = 173 K
0.29 × 0.24 × 0.13 mm

#### Data collection

Bruker SMART APEXII CCD diffractometer
Absorption correction: multi-scan (*SADABS*; Bruker, 2009 ▶) *T*_min_ = 0.903, *T*_max_ = 0.955
16044 measured reflections
4231 independent reflections
3596 reflections with *I* > 2σ(*I*)
*R*_int_ = 0.027

#### Refinement

*R*[*F* ^2^ > 2σ(*F* ^2^)] = 0.046
*wR*(*F* ^2^) = 0.132
*S* = 1.06
4231 reflections
211 parameters
H-atom parameters constrained
Δρ_max_ = 0.85 e Å^−3^
Δρ_min_ = −0.46 e Å^−3^

### 

Data collection: *APEX2* (Bruker, 2009 ▶); cell refinement: *SAINT* (Bruker, 2009 ▶); data reduction: *SAINT*; program(s) used to solve structure: *SHELXS97* (Sheldrick, 2008 ▶); program(s) used to refine structure: *SHELXL97* (Sheldrick, 2008 ▶); molecular graphics: *ORTEP-3* (Farrugia, 1997 ▶) and *DIAMOND* (Brandenburg, 1998 ▶); software used to prepare material for publication: *SHELXL97*.

## Supplementary Material

Crystal structure: contains datablock(s) global, I. DOI: 10.1107/S1600536812008525/lr2052sup1.cif

Structure factors: contains datablock(s) I. DOI: 10.1107/S1600536812008525/lr2052Isup2.hkl

Supplementary material file. DOI: 10.1107/S1600536812008525/lr2052Isup3.cml

Additional supplementary materials:  crystallographic information; 3D view; checkCIF report

## Figures and Tables

**Table 1:** Hydrogen-bond geometry (Å, °) *Cg* is the centroid of the C2–C7 benzene ring.

*D*—H⋯*A*	*D*—H	H⋯*A*	*D*⋯*A*	*D*—H⋯*A*
C5—H5⋯O3^i^	0.95	2.42	3.321 (2)	159
C12—H12*A*⋯O2^ii^	0.98	2.42	3.375 (2)	165
C16—H16⋯*Cg*^iii^	0.95	2.71	3.646 (2)	170

Symmetry codes: (i) 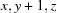; (ii) 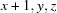; (iii) 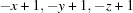.

**Fig. 1.**
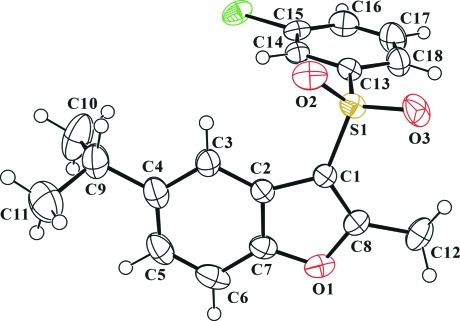
The molecular structure of the title compound. Displacement ellipsoids are drawn at the 50% probability level.

**Fig. 2.**
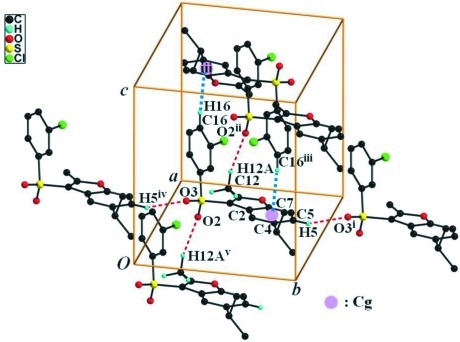
A view of the C—H···O and C—H···π interactions (dotted lines) in the crystal structure of the title compound. H atoms non-participating in hydrogen-bonding were omitted for clarity. [ Symmetry codes: (i) x, y + 1, z; (ii) x + 1, y, z; (iii) - x + 1, - y + 1, - z + 1 (iv) x, y - 1, z; (v) x - 1, y, z.]

## supplementary crystallographic information

### Comment

As a part of our ongoing study of 5-isopropyl-2-methyl-1-benzofuran derivatives containing either 3-(4-fluorophenylsulfonyl) (Choi *et al.*, 2010) or 3-(4-chlorophenylsufonyl) (Choi *et al.*, 2011) substituents, we report herein the crystal structure of the title compound.

In the title molecule (Fig. 1), the benzofuran unit is essentially planar, with a mean deviation of 0.006 (1) Å from the least-squares plane defined by the nine constituent atoms. The dihedral angle between the 3-chlorobenzene ring and the mean plane of the benzofuran fragment is 82.04 (5)°. The crystal packing is stabilized by weak intermolecular C—H···O hydrogen bonds (Fig. 2 & Table 1). The crystal packing is further stabilized by intermolecular C—H···π interactions (Fig. 2 & Table 1, Cg is the centroid of the C2–C7 benzene ring).

### Experimental

77% 3-Chloroperoxybenzoic acid (381 mg, 1.7 mmol) was added in small portions to a stirred solution of 3-(3-chlorophenylsulfanyl)-5-isopropyl-2-methyl-1-benzofuran (279 mg, 0.8 mmol) in dichloromethane (30 mL) at 273 K. After being stirred at room temperature for 8h, the mixture was washed with saturated sodium bicarbonate solution and the organic layer was separated, dried over magnesium sulfate, filtered and concentrated at reduced pressure. The residue was purified by column chromatography (hexane–ethyl acetate, 4:1 v/v) to afford the title compound as a colorless solid [yield 70%, m.p. 357–358 K; *R*_f_ = 0.51 (hexane–ethyl acetate, 4:1 v/v)]. Single crystals suitable for X-ray diffraction were prepared by slow evaporation of a solution of the title compound in diisopropyl ether at room temperature.

### Refinement

All H atoms were positioned geometrically and refined using a riding model, with C—H = 0.95 Å for aryl and 0.98 Å for methyl H atoms. *U*_iso_(H) = 1.2*U*_eq_(C) for aryl and 1.5*U*_eq_(C) for methyl H atoms.

### Crystal data

**Table:**

C_18_H_17_ClO_3_S	*Z* = 2
*M**_r_* = 348.83	*F*(000) = 364
Triclinic, *P*1	*D*_x_ = 1.368 Mg m^−^^3^
Hall symbol: -P 1	Mo *K*α radiation, λ = 0.71073 Å
*a* = 7.1700 (2) Å	Cell parameters from 7011 reflections
*b* = 9.9400 (2) Å	θ = 2.6–28.4°
*c* = 12.2508 (3) Å	µ = 0.36 mm^−^^1^
α = 83.484 (1)°	*T* = 173 K
β = 77.907 (1)°	Block, colourless
γ = 85.707 (1)°	0.29 × 0.24 × 0.13 mm
*V* = 847.05 (4) Å^3^	

### Data collection

**Table:**

Bruker SMART APEXII CCD diffractometer	4231 independent reflections
Radiation source: rotating anode	3596 reflections with *I* > 2σ(*I*)
Graphite multilayer monochromator	*R*_int_ = 0.027
Detector resolution: 10.0 pixels mm^-1^	θ_max_ = 28.4°, θ_min_ = 1.7°
φ and ω scans	*h* = −9→9
Absorption correction: multi-scan (*SADABS*; Bruker, 2009)	*k* = −13→13
*T*_min_ = 0.903, *T*_max_ = 0.955	*l* = −16→16
16044 measured reflections	

### Refinement

**Table:**

Refinement on *F*^2^	Primary atom site location: structure-invariant direct methods
Least-squares matrix: full	Secondary atom site location: difference Fourier map
*R*[*F*^2^ > 2σ(*F*^2^)] = 0.046	Hydrogen site location: difference Fourier map
*wR*(*F*^2^) = 0.132	H-atom parameters constrained
*S* = 1.06	*w* = 1/[σ^2^(*F*_o_^2^) + (0.0688*P*)^2^ + 0.4272*P*] where *P* = (*F*_o_^2^ + 2*F*_c_^2^)/3
4231 reflections	(Δ/σ)_max_ < 0.001
211 parameters	Δρ_max_ = 0.85 e Å^−^^3^
0 restraints	Δρ_min_ = −0.46 e Å^−^^3^

### Special details

**Table:**

Geometry. All esds (except the esd in the dihedral angle between two l.s. planes) are estimated using the full covariance matrix. The cell esds are taken into account individually in the estimation of esds in distances, angles and torsion angles; correlations between esds in cell parameters are only used when they are defined by crystal symmetry. An approximate (isotropic) treatment of cell esds is used for estimating esds involving l.s. planes.
Refinement. Refinement of F^2^ against ALL reflections. The weighted R-factor wR and goodness of fit S are based on F^2^, conventional R-factors R are based on F, with F set to zero for negative F^2^. The threshold expression of F^2^ > 2sigma(F^2^) is used only for calculating R-factors(gt) etc. and is not relevant to the choice of reflections for refinement. R-factors based on F^2^ are statistically about twice as large as those based on F, and R- factors based on ALL data will be even larger.

### Fractional atomic coordinates and isotropic or equivalent isotropic displacement parameters (Å^2^)

**Table:**

	*x*	*y*	*z*	*U*_iso_*/*U*_eq_	
Cl1	0.22055 (7)	0.49026 (6)	0.63396 (4)	0.04596 (16)	
S1	0.34765 (7)	0.31464 (4)	0.22246 (3)	0.03083 (13)	
O1	0.71701 (19)	0.58220 (14)	0.07545 (10)	0.0376 (3)	
O2	0.1494 (2)	0.35118 (15)	0.22738 (12)	0.0418 (3)	
O3	0.4313 (3)	0.19619 (14)	0.17075 (12)	0.0470 (4)	
C1	0.4757 (2)	0.45364 (16)	0.16032 (13)	0.0269 (3)	
C2	0.4074 (3)	0.59381 (16)	0.16760 (13)	0.0287 (3)	
C3	0.2339 (3)	0.66147 (18)	0.21091 (15)	0.0350 (4)	
H3	0.1263	0.6116	0.2471	0.042*	
C4	0.2210 (3)	0.8016 (2)	0.20043 (17)	0.0424 (5)	
C5	0.3805 (4)	0.8721 (2)	0.14704 (18)	0.0485 (5)	
H5	0.3705	0.9684	0.1413	0.058*	
C6	0.5538 (4)	0.8085 (2)	0.10175 (18)	0.0476 (5)	
H6	0.6607	0.8585	0.0645	0.057*	
C7	0.5627 (3)	0.66778 (19)	0.11380 (14)	0.0346 (4)	
C8	0.6615 (3)	0.45245 (18)	0.10554 (14)	0.0314 (4)	
C9	0.0286 (4)	0.8735 (2)	0.2472 (2)	0.0510 (5)	
H9	−0.0715	0.8055	0.2589	0.061*	
C10	0.0305 (5)	0.9199 (4)	0.3600 (2)	0.0800 (10)	
H10A	0.1277	0.9867	0.3513	0.120*	
H10B	0.0601	0.8419	0.4112	0.120*	
H10C	−0.0952	0.9612	0.3907	0.120*	
C11	−0.0257 (5)	0.9892 (3)	0.1681 (3)	0.0735 (8)	
H11A	−0.1572	1.0223	0.1965	0.110*	
H11B	−0.0162	0.9582	0.0939	0.110*	
H11C	0.0608	1.0626	0.1622	0.110*	
C12	0.8097 (3)	0.3445 (2)	0.07479 (18)	0.0445 (5)	
H12A	0.8950	0.3338	0.1285	0.067*	
H12B	0.8836	0.3690	−0.0008	0.067*	
H12C	0.7490	0.2590	0.0763	0.067*	
C13	0.3857 (3)	0.29535 (17)	0.36169 (14)	0.0301 (3)	
C14	0.2933 (2)	0.38686 (17)	0.43515 (14)	0.0300 (3)	
H14	0.2060	0.4559	0.4127	0.036*	
C15	0.3317 (3)	0.3750 (2)	0.54204 (14)	0.0337 (4)	
C16	0.4560 (3)	0.2732 (2)	0.57601 (17)	0.0448 (5)	
H16	0.4809	0.2661	0.6497	0.054*	
C17	0.5430 (4)	0.1826 (2)	0.50199 (19)	0.0509 (5)	
H17	0.6268	0.1117	0.5254	0.061*	
C18	0.5105 (3)	0.1929 (2)	0.39347 (17)	0.0425 (4)	
H18	0.5727	0.1309	0.3422	0.051*	

### Atomic displacement parameters (Å^2^)

**Table:**

	*U*^11^	*U*^22^	*U*^33^	*U*^12^	*U*^13^	*U*^23^
Cl1	0.0426 (3)	0.0637 (3)	0.0318 (2)	−0.0110 (2)	0.00138 (19)	−0.0171 (2)
S1	0.0390 (2)	0.0288 (2)	0.0274 (2)	−0.00753 (17)	−0.00952 (17)	−0.00579 (15)
O1	0.0365 (7)	0.0478 (8)	0.0290 (6)	−0.0133 (6)	−0.0028 (5)	−0.0056 (6)
O2	0.0366 (7)	0.0537 (8)	0.0395 (7)	−0.0132 (6)	−0.0144 (6)	−0.0029 (6)
O3	0.0747 (11)	0.0293 (6)	0.0396 (7)	−0.0061 (7)	−0.0112 (7)	−0.0126 (6)
C1	0.0312 (8)	0.0283 (7)	0.0227 (7)	−0.0026 (6)	−0.0075 (6)	−0.0047 (6)
C2	0.0369 (9)	0.0279 (8)	0.0223 (7)	−0.0019 (7)	−0.0080 (6)	−0.0036 (6)
C3	0.0419 (10)	0.0341 (9)	0.0295 (8)	0.0035 (7)	−0.0087 (7)	−0.0057 (7)
C4	0.0611 (13)	0.0339 (9)	0.0339 (9)	0.0078 (9)	−0.0154 (9)	−0.0060 (7)
C5	0.0783 (16)	0.0285 (9)	0.0412 (11)	0.0013 (10)	−0.0184 (11)	−0.0053 (8)
C6	0.0684 (15)	0.0405 (10)	0.0359 (10)	−0.0230 (10)	−0.0118 (10)	0.0035 (8)
C7	0.0452 (10)	0.0355 (9)	0.0241 (8)	−0.0089 (8)	−0.0070 (7)	−0.0032 (6)
C8	0.0336 (9)	0.0399 (9)	0.0230 (7)	−0.0023 (7)	−0.0084 (6)	−0.0084 (7)
C9	0.0598 (14)	0.0427 (11)	0.0509 (12)	0.0112 (10)	−0.0137 (11)	−0.0104 (9)
C10	0.0746 (19)	0.110 (3)	0.0598 (16)	0.0405 (18)	−0.0238 (15)	−0.0397 (17)
C11	0.0756 (19)	0.0659 (17)	0.080 (2)	0.0106 (15)	−0.0291 (16)	0.0048 (15)
C12	0.0342 (10)	0.0599 (13)	0.0421 (10)	0.0094 (9)	−0.0111 (8)	−0.0176 (9)
C13	0.0352 (9)	0.0282 (8)	0.0275 (8)	−0.0057 (7)	−0.0076 (7)	−0.0010 (6)
C14	0.0290 (8)	0.0333 (8)	0.0284 (8)	−0.0054 (7)	−0.0060 (6)	−0.0035 (6)
C15	0.0316 (9)	0.0432 (10)	0.0258 (8)	−0.0117 (7)	−0.0018 (7)	−0.0031 (7)
C16	0.0506 (12)	0.0543 (12)	0.0317 (9)	−0.0072 (10)	−0.0153 (9)	0.0032 (8)
C17	0.0606 (14)	0.0468 (12)	0.0465 (12)	0.0093 (10)	−0.0224 (11)	0.0039 (9)
C18	0.0513 (12)	0.0353 (9)	0.0404 (10)	0.0065 (8)	−0.0114 (9)	−0.0040 (8)

### Geometric parameters (Å, º)

**Table:**

Cl1—C15	1.7307 (19)	C9—C10	1.509 (4)
S1—O2	1.4308 (15)	C9—H9	1.0000
S1—O3	1.4325 (14)	C10—H10A	0.9800
S1—C1	1.7290 (17)	C10—H10B	0.9800
S1—C13	1.7700 (18)	C10—H10C	0.9800
O1—C8	1.366 (2)	C11—H11A	0.9800
O1—C7	1.379 (2)	C11—H11B	0.9800
C1—C8	1.360 (2)	C11—H11C	0.9800
C1—C2	1.448 (2)	C12—H12A	0.9800
C2—C7	1.386 (3)	C12—H12B	0.9800
C2—C3	1.400 (2)	C12—H12C	0.9800
C3—C4	1.382 (3)	C13—C18	1.382 (3)
C3—H3	0.9500	C13—C14	1.386 (2)
C4—C5	1.389 (3)	C14—C15	1.384 (2)
C4—C9	1.534 (3)	C14—H14	0.9500
C5—C6	1.387 (3)	C15—C16	1.385 (3)
C5—H5	0.9500	C16—C17	1.374 (3)
C6—C7	1.387 (3)	C16—H16	0.9500
C6—H6	0.9500	C17—C18	1.388 (3)
C8—C12	1.475 (3)	C17—H17	0.9500
C9—C11	1.501 (3)	C18—H18	0.9500
			
O2—S1—O3	119.69 (9)	C9—C10—H10A	109.5
O2—S1—C1	107.76 (8)	C9—C10—H10B	109.5
O3—S1—C1	109.52 (9)	H10A—C10—H10B	109.5
O2—S1—C13	107.50 (8)	C9—C10—H10C	109.5
O3—S1—C13	107.43 (9)	H10A—C10—H10C	109.5
C1—S1—C13	103.79 (8)	H10B—C10—H10C	109.5
C8—O1—C7	107.30 (14)	C9—C11—H11A	109.5
C8—C1—C2	107.79 (15)	C9—C11—H11B	109.5
C8—C1—S1	126.80 (14)	H11A—C11—H11B	109.5
C2—C1—S1	125.15 (13)	C9—C11—H11C	109.5
C7—C2—C3	119.77 (16)	H11A—C11—H11C	109.5
C7—C2—C1	104.46 (16)	H11B—C11—H11C	109.5
C3—C2—C1	135.75 (17)	C8—C12—H12A	109.5
C4—C3—C2	119.30 (19)	C8—C12—H12B	109.5
C4—C3—H3	120.3	H12A—C12—H12B	109.5
C2—C3—H3	120.3	C8—C12—H12C	109.5
C3—C4—C5	119.2 (2)	H12A—C12—H12C	109.5
C3—C4—C9	118.4 (2)	H12B—C12—H12C	109.5
C5—C4—C9	122.43 (19)	C18—C13—C14	121.69 (17)
C6—C5—C4	123.05 (18)	C18—C13—S1	119.55 (14)
C6—C5—H5	118.5	C14—C13—S1	118.73 (14)
C4—C5—H5	118.5	C15—C14—C13	118.22 (17)
C7—C6—C5	116.5 (2)	C15—C14—H14	120.9
C7—C6—H6	121.8	C13—C14—H14	120.9
C5—C6—H6	121.8	C14—C15—C16	121.22 (18)
O1—C7—C2	110.48 (15)	C14—C15—Cl1	118.77 (15)
O1—C7—C6	127.30 (18)	C16—C15—Cl1	120.01 (15)
C2—C7—C6	122.21 (19)	C17—C16—C15	119.26 (19)
C1—C8—O1	109.96 (15)	C17—C16—H16	120.4
C1—C8—C12	134.29 (18)	C15—C16—H16	120.4
O1—C8—C12	115.74 (16)	C16—C17—C18	121.1 (2)
C11—C9—C10	111.0 (2)	C16—C17—H17	119.5
C11—C9—C4	112.9 (2)	C18—C17—H17	119.5
C10—C9—C4	110.2 (2)	C13—C18—C17	118.53 (19)
C11—C9—H9	107.5	C13—C18—H18	120.7
C10—C9—H9	107.5	C17—C18—H18	120.7
C4—C9—H9	107.5		
			
O2—S1—C1—C8	−156.03 (15)	S1—C1—C8—O1	−175.31 (11)
O3—S1—C1—C8	−24.32 (18)	C2—C1—C8—C12	178.02 (19)
C13—S1—C1—C8	90.16 (16)	S1—C1—C8—C12	3.6 (3)
O2—S1—C1—C2	30.48 (16)	C7—O1—C8—C1	0.90 (19)
O3—S1—C1—C2	162.19 (14)	C7—O1—C8—C12	−178.25 (15)
C13—S1—C1—C2	−83.33 (16)	C3—C4—C9—C11	−135.1 (2)
C8—C1—C2—C7	0.54 (18)	C5—C4—C9—C11	44.1 (3)
S1—C1—C2—C7	175.07 (13)	C3—C4—C9—C10	100.2 (3)
C8—C1—C2—C3	178.83 (19)	C5—C4—C9—C10	−80.7 (3)
S1—C1—C2—C3	−6.6 (3)	O2—S1—C13—C18	141.75 (16)
C7—C2—C3—C4	−0.7 (3)	O3—S1—C13—C18	11.71 (19)
C1—C2—C3—C4	−178.83 (18)	C1—S1—C13—C18	−104.25 (17)
C2—C3—C4—C5	0.0 (3)	O2—S1—C13—C14	−40.32 (16)
C2—C3—C4—C9	179.19 (17)	O3—S1—C13—C14	−170.36 (14)
C3—C4—C5—C6	0.9 (3)	C1—S1—C13—C14	73.68 (15)
C9—C4—C5—C6	−178.2 (2)	C18—C13—C14—C15	1.1 (3)
C4—C5—C6—C7	−1.1 (3)	S1—C13—C14—C15	−176.74 (13)
C8—O1—C7—C2	−0.54 (19)	C13—C14—C15—C16	−1.2 (3)
C8—O1—C7—C6	−179.70 (19)	C13—C14—C15—Cl1	178.92 (13)
C3—C2—C7—O1	−178.62 (15)	C14—C15—C16—C17	0.1 (3)
C1—C2—C7—O1	0.00 (19)	Cl1—C15—C16—C17	−179.99 (17)
C3—C2—C7—C6	0.6 (3)	C15—C16—C17—C18	1.0 (4)
C1—C2—C7—C6	179.21 (17)	C14—C13—C18—C17	0.0 (3)
C5—C6—C7—O1	179.36 (18)	S1—C13—C18—C17	177.84 (17)
C5—C6—C7—C2	0.3 (3)	C16—C17—C18—C13	−1.1 (4)
C2—C1—C8—O1	−0.90 (19)		

### Hydrogen-bond geometry (Å, º)

Cg is the centroid of the C2–C7 benzene ring.

**Table:**

*D*—H···*A*	*D*—H	H···*A*	*D*···*A*	*D*—H···*A*
C5—H5···O3^i^	0.95	2.42	3.321 (2)	159
C12—H12*A*···O2^ii^	0.98	2.42	3.375 (2)	165
C16—H16···*Cg*^iii^	0.95	2.71	3.646 (2)	170

Symmetry codes: (i) *x*, *y*+1, *z*; (ii) *x*+1, *y*, *z*; (iii) −*x*+1, −*y*+1, −*z*+1.
